# Torulene and torularhodin: “new” fungal carotenoids for industry?

**DOI:** 10.1186/s12934-018-0893-z

**Published:** 2018-03-27

**Authors:** Anna M. Kot, Stanisław Błażejak, Iwona Gientka, Marek Kieliszek, Joanna Bryś

**Affiliations:** 1 Department of Biotechnology, Microbiology and Food Evaluation, Faculty of Food Sciences, Warsaw University of Life Sciences, Nowoursynowska 159C, 02-776 Warsaw, Poland; 2 Department of Chemistry, Faculty of Food Sciences, Warsaw University of Life Sciences, Nowoursynowska 159C, 02-776 Warsaw, Poland

**Keywords:** Microbial carotenoids, Yeast, *Rhodotorula*, *Sporobolomyces*, Dyes

## Abstract

Torulene and torularhodin represent the group of carotenoids and are synthesized by yeasts and fungi. The most important producers of these two compounds include yeasts of *Rhodotorula* and *Sporobolomyces* genera. The first reports confirming the presence of torulene and torularhodin in the cells of microorganisms date to the 1930s and 1940s; however, only in the past few years, the number of works describing the properties of these compounds increased. These compounds have strong anti-oxidative and anti-microbial properties, and thus may be successfully used as food, feedstock, and cosmetics additives. In addition, tests performed on rats and mice showed that both torulene and torularhodin have anti-cancerous properties. In order to commercialize the production of these two carotenoids, it is necessary to obtain highly efficient yeast strains, for example, via mutagenization and optimization of cultivation conditions. Further studies on the activity of torulene and torularhodin on the human body are also needed.

## Background

Carotenoids are a group of compounds commonly present in nature. They are characterized by a yellow, orange, or red color and occur naturally in fruits, vegetables, algae, fish, eggs, and oil [1]. Nearly 750 carotenoids have been identified, including 50 found in food consumed by humans [2]. These compounds are the main source of vitamin A in the human diet, and they have health-promoting properties. Carotenoids reinforce the immune system of the body, accelerate the healing of wounds [3], and counteract eye conditions, such as cataracts [4] or age-related macular degeneration [5]. Carotenoid supplements are commonly used as agents protecting the skin against harmful ultraviolet radiation [6]. Carotenoids may also be used in cancer prevention owing to their anti-oxidative properties [7]. The human body cannot biosynthesize these compounds, thus they have to be supplemented in food. Carotenoids are widely used in the industry, i.e., as additives to food, diet supplements, and cosmetics. They are also an important ingredient of feedstock for poultry, fish, and mollusks. Currently, the greatest industrial importance is attached to β-carotene, astaxanthin, lutein, zeaxanthin, and canthaxanthin [8]. Because of their valuable properties, interest in other carotenoids has also increased recently. Such compounds include torulene and torularhodin. Their structure, properties, and potential industrial applications are described in this work. In addition, their sources, biosynthetic pathway, methods of extraction and analysis, and a recommended production method are presented.

## Structure and sources of torulene and torularhodin

Carotenoids are organic compounds and belong to 40-carbon terpenoids. The structure of the isoprene chain allowed to separate two groups of these compounds, and the presence of oxygen in the molecule provides the dividing criterion. Carotenes include only carbon and hydrogen atoms in their molecules, whereas xanthophylls have at least one additional oxygen atom in the molecule [9]. It is an element of a carboxy, hydroxy, carbonyl, or hydroxymethyl group. The presence of such functional groups renders xanthophylls more polar than carotenes [10]. Carotenes include, for instance, β-carotene and torulene, whereas xanthophylls include astaxanthin and canthaxanthin [9]. Due to the presence of a carboxy group in the torularhodin molecule, this compound may be classified as one of the xanthophylls.

**Fig. 1:**
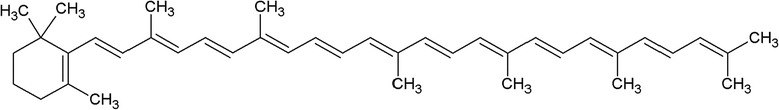
Structural formula of torulene [17]

**Fig. 2:**
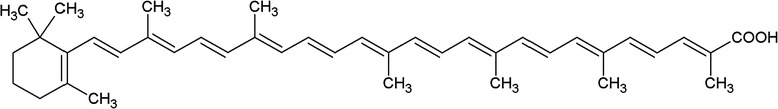
Structural formula of torularhodin [17]

Torulene (C_40_H_54_, 3′,4′‐didehydro‐β,ψ‐carotene, Fig. 1) and torularhodin (C_40_H_52_O_2_, 3′,4′‐didehydro‐β,ψ‐caroten‐16′‐oic acid, Fig. 2) have one β-ionone ring connected to a polyene chain [11]. Due to the presence of thirteen double bonds, torulene and torularhodin have strong anti-oxidative properties [12–14]. It has been reported [15] that maximum absorption for torulene is observed at 460, 484 and 518 nm in petroleum ether. Torularhodin absorbed maximally at 465, 492 and 523 nm in petroleum ether. Depending on their concentration, they have a rosy-red color [16].

The first literature reports confirming the presence of torularhodin were released in the 1930s and were related to dyes extracted from biomass of yeasts of the *Rhodotorula* genus [18]. In 1946, Bonner et al. [19] found torulene in the cells of *Rhodotorula rubra* yeasts. Since then, the presence of torulene and torularhodin in the cells of yeast and fungi has been described; however, only the last decade observed an increase in the number of studies related to possibilities of synthesis of these compounds [15].

Torulene is synthesized by fungi of the genera *Cystofilobasidium* [11], *Dioszegia* [20, 21], *Neurospora* [22], *Rhodotorula* [23], *Rhodosporidium* [24], *Sporidiobolus* [25, 26], and *Sporobolomyces* [27–29]. Torularhodin is synthesized by fungi of the genera *Cystofilobasidium* [11], *Rhodotorula* [30, 31], *Rhodosporidium* [32], *Sporidiobolus* [33] and *Sporobolomyces* [27–29, 34]. Although these genera include a large number of species, only a few of these can accumulate significant amounts of these carotenoids. *Cystofilobasidium infirmominiatum* and *Cystofilobasidium capitatum* are included in the class of Tremellomycetes, *Neurospora crassa* is included in the class of Sordariomycetes, while *Rhodotorula glutinis*, *Rhodotorula mucilaginosa* (syn. *R*. *rubra*), *Rhodosporidium babjevae, Rhodosporidium toruloides Rhodotorula graminis*, *Sporidiobolus pararoseus*, *Sporidiobolus johnsonii Sporobolomyces ruberrimus*, and *Sporobolomyces salmonicolor* are included in the class of Microbotryomycetes [35].

Due to the efficiency of biosynthesis, the most important producers of these two compounds include yeasts of *Rhodotorula*, *Sporidiobolus* and *Sporobolomyces* genera. Levels of torulene and torularhodin contents in the cellular biomass of various yeast strains are presented in Table 1.

**Table 1:** Yield of torulene and torularhodin [mg/L or mg/100 g_d.w._] biosynthesis by various yeast strains

Microorganism	Cultivation medium/conditions	Torulene	Torularhodin	References
mg/100 g_d.w._				
*Rhodotorula glutinis* mutant TL/21	Addition of 0.01 mM bromothymol blue	–	4.9	[36]
*Rhodotorula glutinis* wild strain	Cultivation with white light irradiation	32.2	14.2	[31]
*Rhodotorula glutinis* DBVPG 3853	Grape must concentrate	8.51	72.13	[23]
	Soy flour extract	9.63	70.84	
Mixed culture *Rhodotorula rubra* GED5 and *Kluyveromyces lactis* MP11	Whey	2.69	22.23	[37]
*Rhodotorula mucilaginosa* F-1	25 °C	15.46	7.47	[38]
	31 °C	8.79	17.72	
	Molasses	18.13	2.34	
	Ketchup production waste	21.45	4.67	
*Sporobolomyces salmonicolor* AL_1_	Saccharose	27.37	45.83	[29]
*Sporidiobolus pararoseus* CCTCC M 2010326	Fed-batch cultivation	18.99	–	[39]
*Sporidiobolus johnsonii* DBVPG 7467	Glucose	0.77	4.12	[40]
*Rhodotorula graminis* DBVPG 7021	Glucose	18.23	9.31	[41]
mg/L				
*Rhodotorula* sp. KF-104	YG medium	1.74	1.92	[42]
*Rhodotorula glutinis* mutant 32	Glucose	5	3	[43]
*Rhodotorula glutinis* var. *glutinis*	Wort	0.53	0.16	[44]
*Rhodotorula rubra* ICCF 209	Glucose + 0.1% oleic acid	–	0.31	[45]
	MS3 medium	–	0.71	
	Fructose	–	0.40	
*Rhodotorula rubra* GED8	Glucose	0.87	1.22	[46]
*Rhodotorula rubra* PTCC 5255	Shaken culture	5.09	7.80	[47]
	Cultivation in a biofermentor, irradiation at 1780 lx	4.48	35.59	
*Rhodotorula mucilaginosa* RCL-11	Control	0.82 mg/L	2.24 mg/L	[48]
	+ CuSO_4_	2.33 mg/L	5.37 mg/L	
	+ H_2_O_2_	8.21 mg/L	5.22 mg/L	
	+ CuSO_4_ + H_2_O_2_	5.60 mg/L	4.03 mg/L	
*Rhodotorula mucilaginosa* 108	YM medium	0.31 mg/L	0.065 mg/L	[15]
*Rhodotorula graminis* 125		0.19 mg/L	–	
*Sporobolomyces* sp.		0.07 mg/L	0.01 mg/L	
*Sporobolomyces ruberrimus* H110	Technical glycerin, pH 6.0	–	31.54 mg/L	[34]
*Sporobolomyces ruberrimus* H110	Raw glycerin	70 mg/L	350 mg/L	[27]

## Role of carotenoids in yeast cells

In microorganisms different functions have been attributed to carotenoids. The primary role of these compounds is protection against the negative influence of reactive forms of oxygen [11] and radiation [30]. Sakaki et al. [13] showed that torularhodin from *R. glutinis* had greater scavenging activity toward peroxyl radicals and inhibits degradation by singlet oxygen more effectively than β-carotene. In another study [12], torularhodin was shown to inhibit peroxidation of lipids, and its inhibitory effect was stronger than that of α-tocopherol at the concentration of 1 μM. Moline et al. [49] tested the photoprotective role of carotenoids in *S. ruberrimus* and *C. capitatum* yeast. Radiation at the wavelengths 320–400 nm (UV-A) causes only indirect damage to DNA, proteins, and lipids [30], while UV-B radiation (280–320 nm) causes damage to DNA by generating two types of mutagenic lesions. To determine the photoprotective role of carotenoids, the authors used pigmented and naturally occurring albino strains of yeast. The tests showed that the pigmented strains were more tolerant to UV-B than the albino strains. In addition, a high content of carotenoids in yeast cells during the stationary growth phase enhanced survival [49]. In their next study [30], it was confirmed that accumulation of torularhodin constitutes an important mechanism that improves the resistance of yeasts to UV-B. It is probable that carotenoids can be associated with modification of membrane permeability and thus increased cellular resistance to oxidation and radiation [50].

## Torulene and torularhodin biosynthesis in yeast cells

**Fig. 3:**
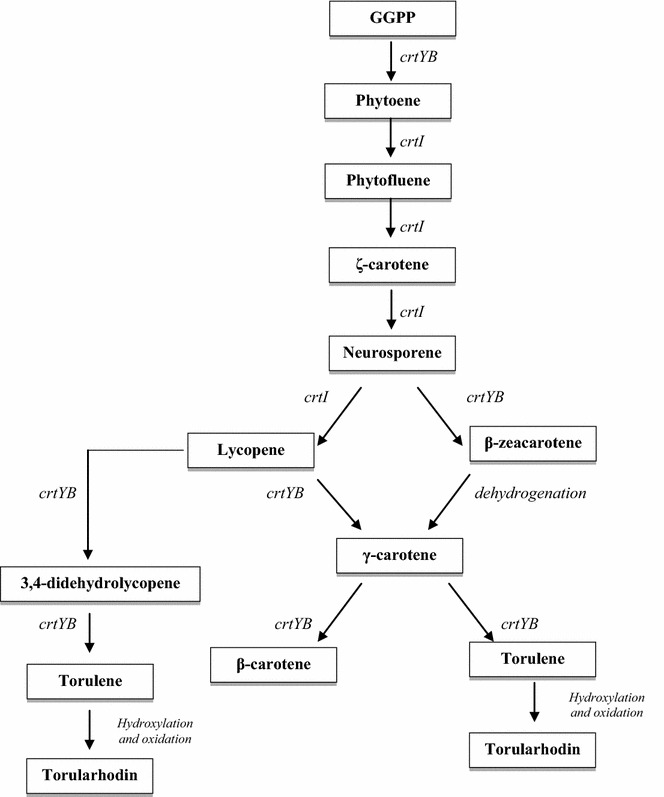
Proposed biosynthesis pathway of torulene and torularhodin in yeast cells from geranylgeranyl pyrophosphate (GGPP). Bifunctional lycopene cyclase/phytoene synthase encoded by *crtYB* gene, phytoene desaturase encoding gene *crtI*. Torularhodin is obtained in transformation of torulene, including hydroxylation and oxidation [26, 53, 54]

Biosynthesis of torulene and torularhodin in microorganism cells includes a range of enzymatic reactions. General pathways for carotenoid biosynthesis have been reviewed by Simpson et al. [51] and later by Goodwin [17]. Firstly, acetyl-CoA is converted to 3-hydroxy-3-methylglutaryl-CoA in the reaction catalyzed by hydroxymethylglutaryl-CoA synthase [EC 2.3.3.10]. Then, 3-hydroxy-3-methylglutaryl-CoA is transformed to mevalonic acid by hydroxymethylglutaryl-CoA reductase [EC 1.1.1.88]. Mevalonic acid is transformed into isopentenyl pyrophosphate (IPP) in several reactions catalyzed by specific kinases [EC 2.7.1.36, EC 2.7.4.2] and diphosphomevalonate decarboxylase [EC 4.1.1.33]. Isopentenyl pyrophosphate is subsequently transformed into dimethylallyl pyrophosphate (DMAPP) via isomerization. The next step, an addition reaction of three IPP molecules, leads to the formation of geranylgeranyl pyrophosphate (GGPP). Condensation of two molecules of this compound leads to the formation of phytoene, and it is catalyzed by phytoene synthase [EC 2.5.1.32]. The next reactions are catalyzed by phytoene desaturase [EC 1.3.99.28-31]. This enzyme is involved in neurosporene formation and catalyzes up to three desaturation steps. Neurosporene may be transformed into lycopene or β-zeacarotene [17, 51, 52]. Lycopene cyclization or β-zeacarotene dehydrogenation leads to the formation of γ-carotene [52]. The γ-carotene molecule provides a precursor for the biosynthesis of β-carotene and torulene, whereas torularhodin is obtained in a further transformation of torulene, including hydroxylation and oxidation (Fig. 3) [53].

According to the study by Li et al. [26], torulene can also be produced from 3,4-dehydrolycopene. This compound is synthesized from lycopene by phytoene desaturase. The transformation from 3,4-didehydrolycopene to torulene is catalyzed by phytoene synthase/lycopene cyclase AL-2. This type of transformation occurs in *S. pararoseus* yeast. It is also known that 3,4-didehydrolycopene is the precursor of torulene in *N. crassa*.

## Use of molecular and -omics tools to characterize and improve carotenoid biosynthesis by red yeasts

In recent years, the use of molecular and -omics tools in the characterization of red yeast has increased [55]. To date, whole genome sequences of some carotenogenic yeast have been identified, for example *R. glutinis* ATCC 204091 [56], *R. graminis* WP1 [57], *R. mucilaginosa* RIT389 [58] *R. toruloides* ATCC 10788 and ATCC 10657 [59], *R. toruloides* MTCC 457 [60], *R. toruloides* CECT1137 [61], and *R. toruloides* CGMCC 2.1609 [62]. Genome sequencing of *R. toruloides* NP11 has led to the identification of two genes that code carotenoid synthesis-related enzymes, phytoene synthase (*PSY1*) and phytoene dehydrogenase (*CRTI*) [63]. Gan et al. [58] identified genomic regions containing the key genes for carotenoid production in *R. mucilaginosa*. The genes coding for phytoene synthase (*crtB*), lycopene cyclase (*crtY*), and phytoene desaturase (*crtI*) were located in relatively close proximity to one another. Gene coding for the enzyme geranyl pyrophosphate synthase is located on separate contigs. Another study [64] identified a set of genes involved in different steps of carotenogenesis in *R. mucilaginosa*. It was shown that genes coding for 3-hydroxy-3-methylglutaryl-CoA reductase and mevalonate kinase are induced during exponential growth phase. This trend was not observed for phytoene synthase/lycopene cyclase and phytoene dehydrogenase encoding genes. It was also found that the transcript levels of genes coding for carotenoid dioxygenase, superoxide dismutase and catalase A increased during the accumulation of carotenoids. Li et al. [26] observed that expression of genes in *S. pararoseus* NGR encoding a phytoene desaturase (*crtI*) and lycopene cyclase and phytoene synthase (*crtB*) were 5.2- and 2.5-fold higher upon exposure to NaCl. Under these conditions, the production of 3,4-didehydrolycopene increases, thus resulting in a subsequent increase in torulene and torularhodin bioproduction.

Genetic engineering tools also have the potential to increase the efficiency of carotenoid biosynthesis by yeast. Wang et al. [65] used chemical–physical mutagenesis for modification of a strain of the red yeast *R. mucilaginosa* KC8, which synthesized mainly β-carotene and torularhodin. After mutagenesis, *R. mucilaginosa* K4 synthesized 67% more carotenoids (14.47 mg/L) than parental strain KC8 (8.67 mg/L). To further enhance carotenoid production, gene HMG1 encoding the 3-hydroxy-3-methylglutaryl coenzyme A (HMG-CoA) reductase was overexpressed in *R. mucilaginosa* K4. The carotenoid production of HMG1-gene-overexpression transformant G1 reached 16.98 mg/L. In the next step, ketoconazole (ergosterol synthesis inhibitor) was added to the cultivation medium at a concentration of 28 mg/L. The carotenoid production of transformant G1 reached 19.14 mg/L, and this was 121% higher than in *R. mucilaginosa* KC8.

## Factors affecting the torulene and torularhodin biosynthesis in yeast cells

The yield of carotenoid biosynthesis and the percentage levels of torulene and torularhodin do not have constant values. The efficiency of this process is primarily influenced by the composition of the medium and the cultivation parameters. The content of individual carotenoid fractions also depends to a large extent on the yeast strain (Table 1).

One of the most important factors that significantly regulates the process of carotenogenesis is the type and content of compounds that constitute sources of carbon and nitrogen. The optimal type of these compounds should be determined individually for each yeast strain [66–69]. Buzzini and Martini [23] cultivated *R. glutinis* yeasts in media containing various carbon sources. The highest total level of carotenoids (915.4 µg/g_d.w._) was obtained in the biomass of yeasts from cultivation in a medium with concentrated grape must. In this case, the highest amount of torulene (85.1 µg/g_d.w._) and torularhodin (721.3 µg/g_d.w._) was also obtained, and their ratio reached 9.3:78.8. The remaining part included β-carotene (9.2%). However, in the medium with glucose syrup, the yield of carotenoid was nearly fourfold less (240.0 µg/g_d.w._), and the ratio of torulene and torularhodin was 10.8:85.0. Under these conditions, the *R. glutinis* yeast was unable to biosynthesize β-carotene.

The next required nutrient that must be present in the medium is nitrogen. El-Banna et al. [67] found that selection of the type of nitrogen compounds significantly influences the yields of torulene and torularhodin biosynthesized by *R. glutinis* yeasts. The highest yield of torulene (243 µg/g_d.w._) was observed after cultivation in a medium with peptone. In these conditions yeast was unable to synthesize torularhodin. The highest level of this compound (20.2 µg/g_d.w._) was obtained in the biomass of yeasts cultivated in a medium containing ammonium sulfate.

Selection of the appropriate concentrations of microelements in the cultivation medium is another factor required in order to achieve high yields of carotenoid biosynthesis [43]. The presence of some metal ions can stimulate or inhibit cellular enzymes participating in production of carotenoid compounds [70]. Experiments performed using *R. graminis* yeast also proved that the content of some trace elements in the cultivation medium influenced the profile of carotenoid. For example, the simultaneous presence of zinc and manganese ions in the medium completely inhibited torulene and torularhodin production [41].

Yeasts of *Rhodotorula* and *Sporobolomyces* genera are aerobic microorganisms, and thus adequate aeration is necessary in order to obtain high biosynthetic yields of torulene and torularhodin. Simova et al. [71] cultivated a mixture of *R. rubra* yeasts and *Lactobacillus casei* subsp. *casei* bacteria. The authors observed that the increase in aeration rate from 0.4–1.3 to 1.6 L/L min led to a decrease in the produced torularhodin from 44.0 to 29%, whereas the levels of torulene remained relatively constant (9.5–11.0%). The content of β-carotene increased, from 42.0 to 60.0%.

Cultivation temperature is an important parameter influencing the amount of carotenoid compounds produced by yeasts. The influence of temperature is correlated to the activity of enzymes participating in the biosynthesis of carotenoids and in their regulation [52]. Temperature influences not only the general amount of produced carotenoids, but also the relative ratio of β-carotene, torulene, and torularhodin levels. Increased synthesis of β-carotene by yeast promotes a lower temperature. In the case of torulene and torularodine, the situation is the opposite. The increase in their production takes place at higher temperatures [51, 72]. It is probable that at low temperature enzymes involved in the biosynthesis of torulene are less active, and the content of β-carotene increases [73]. Frengova et al. [72] cultivated co-culture of *R. rubra* and *Lactobacillus helveticus* at 20 and 35 °C. In the first case, β-carotene, torulene, and torularhodin content amounted to 19.0, 22.8, and 56.0%, respectively. At 35 °C, total amount of β-carotene and torulene was low (9.6 and 9.0%), while the content of torularhodin increased significantly (78.3%).

The active acidity of the culture medium also significantly affects the yield and profile of carotenoids synthesized by microorganisms. Cheng and Yang [38] studied the influence of initial pH of the medium (from 4 to 7) on the content and profile of carotenoids synthesized by *R. mucilaginosa* R-1 yeasts. The initial pH of the medium had the strongest influence on the content of β-carotene and torularhodin. After cultivation in a YM medium with the initial pH of 4.0, torularhodin content was 20.1%, which increased to 36.0% in a medium at pH 7.0. A reverse trend was observed for β-carotene, its amount decreased under these conditions from 41.1 to 13.6%.

Addition to the culture medium of some chemical compounds and modification of culture conditions may induce the process of carotenoid biosynthesis in yeast cells [70]. In order to cope with the harmful effects of stress, microorganisms have developed defensive reactions to quickly repair damage and further protect the cell. It has also been found [74] that yeasts subjected to a mild form of stress can after some time tolerate higher doses of a stress factor. Such environmental factors include irradiation, increased osmotic pressure, the presence of reactive oxygen species and also some organic solvents [70, 75, 76].

Increasing the content of carotenoids during exposure to irradiation results from the higher expression of genes coding for the production of enzymes involved in the carotenoid biosynthetic pathway. This is a natural mechanism of cell response to adverse environmental conditions [70]. Sakaki et al. [31] determined the influence of white light on carotenoid biosynthesis by *R. glutinis* no. 21 yeasts. They concluded that irradiation of the cultivation intensified the production of all carotenoids, in particular of torularhodin. The amount of this compound showed an almost twofold increase, from 7.9 to 14.2 mg/100 g_d.w._.

Addition of various chemicals, such as ethanol, methanol, isopropanol, or glycol to the cultivation medium may also influence the biosynthesis of torulene and torularhodin by yeasts. For example, supplementation of the cultivation medium with 2% ethanol stimulated the biosynthesis of β-carotene and torulene by *R. glutinis* yeasts, whereas torularhodin synthesis was limited under these conditions [73].

## Extraction and analysis of torulene and torularhodin

Carotenoids present in yeast cells are accumulated mainly in lipid bodies, and thus an appropriate method of cell disintegration should be applied in order to ensure highly efficient extraction of these compounds. However, disintegration and extraction processes must also ensure the stability of carotenoids. Torulene and torularhodin are highly unstable compounds and should be protected from light, heat and oxygen. Carotenoids lose their color during exposure to oxidizing species. This process involves interruption of the conjugated double bonds [77].

Mechanical techniques such as high-pressure homogenization and ball mills are traditionally used for disintegration. However, because of the high costs and long performance time [78], disintegration methods using ultrasound, enzymatic preparations, chemicals such as dimethyl sulfoxide (DMSO), sodium carbonate and acids [79], as well as the use of liquid nitrogen [80] are growing increasingly popular.

Organic solvents such as acetone, petroleum ether, hexane, chloroform, ethanol, and methanol are commonly used in the extraction of carotenoids from microbial cells. These solvents are more often used as mixtures and display synergic activity, additionally damaging the cell wall, thus resulting in significantly increased yields of carotenoid extraction [78]. Park et al. [81] achieved the highest yield of carotenoid extraction from the biomass of *R. glutinis* KCTC 7989 yeasts using a mixture of dimethyl sulfoxide, petroleum ether, and acetone. A combination of these compounds is commonly used in the extraction of carotenoids from biomasses of yeasts synthesizing torulene and torularhodin [82–86]. Carotenoids may also be extracted from microbial cells using plant oils. Mihalcea et al. [77] used sunflower oil for this purpose. Biomass of *R. rubra* yeasts was initially subjected to high-pressure disintegration (five cycles, 1500–2000 bar). After this stage, the cells were suspended in 50 mL of sunflower oil and emulsified in a homogenizing device at 1500 bar. This treatment allowed a pink emulsion to be obtained, containing a water-based part and an oil-based part containing the carotenoids. Ungureanu et al. [87] proposed the use of centrifugal partition extraction (CPE) as a novel method for recovery of torularhodin from *R. rubra* cells. For extraction a mixture of hexane/water with acetone was used as the “bridge solvent”. The compositions of the stationary phase and the mobile phase were 29/71/0 and 2/62/36 (v/v/v), respectively, for hexane/acetone/water. The efficiency of the extraction increased with increased operating flow rate. The authors demonstrated that the extraction yield could reach 91% in a few minutes. The recovery of torularhodin reaches 294 µg/L of culture medium.

Subsequently, the extracted carotenoids should be subjected to separation and purification. Latha and Jeevaratanm [88] used column chromatography for separation of pigments of a crude extract from the yeast *R. glutinis* DFR-PDY. In these conditions, carotenoids were fractioned on magnesium oxide-Hyflo Super Cel by petroleum ether. The major red-colored fraction—torularhodin, was adsorbed on the column, while β-carotene and torulene were eluted by the petroleum ether, ethyl ether and methanol. Torularhodin was eluted with acetic acid–ethyl ether (1:10). Ungureanu and Ferdes [89] reported that extraction with alkaline methanol allows isolation of the torularhodin (acid structure) component from a carotenoid mixture. For the purification, Latha and Jeevaratanm [88] saponified fractions by adding potassium hydroxide-methyl alcohol solution, and next added petroleum ether to the mixture. This solvent was then dried over anhydrous sodium sulfate. For purification of torularhodin produced by *S. ruberrimus* H110, Razavi and Blanchard [90] used a perfusion chromatography work station. Torularhodin was eluted by applying a 150 × 4.6 mm, Symmetry C18 (3.5 µm) column with an acetonitrile–methanol–dichloromethane mixture. After this process, solvents were evaporated and torularhodin was stored in a mixture of hexane and ethyl acetate, at − 18 °C.

Torulene and torularhodin concentrations can be determined based on the spectrometric recording of the extracts on a UV–Vis spectrophotometer [55, 89]. This method is simple, but accurate only when there is one compound in the sample [55]. Carotenoids have specific absorbance spectra, and during analysis of a mixture of these compounds, the absorbance may change depending on the wavelength applied [91].

A new method that allows the measurement of the total carotenoid content in yeast biomass in near real time is multi-parameter flow cytometry. The analysis is performed in vivo, immediately after sampling, which reduces degradation of the carotenoids [55]. Freitas et al. [92] used flow cytometry for determination of total carotenoid content in *R. toruloides* NCYC 921. This strain synthesizes mainly β-carotene, torulene and torularhodin. Based on the obtained results, the authors concluded that flow cytometry can be used in the optimization of yeast carotenoid production, from lab to pilot scales, but this technique gives information only on the total carotenoid content.

A more accurate method for carotenoid quantification involves the uses of reverse-phase high-performance liquid chromatography. This method allows the detection and quantification of the individual carotenoids in a mixture [55]. Currently, the most commonly used system includes a reverse phase system using C_18_ analytical columns [93]. Separation may be performed either by isocratic or a gradient elution, using both organic solvents and their mixtures with water. These compounds may be subsequently identified using spectrophotometric detectors, light-scattering detectors with evaporation, diode-type detectors, refractometric detectors (Table 2), or using mass spectrometry [27, 93]. Razavi and Blanchard [90] determined that the limit of detection for torularhodin is estimated at 9 ng/mL. For separated isometric forms of carotenoids it is necessary to used specific types of cinematographic columns. For example, Shi et al. [25] used an HPLC–DAD coupled with an atmospheric-pressure chemical ionization (APCI) MS method for identification of torulene *cis*/*trans* geometrical isomers isolated from *S. pararoseus*. The torulene fraction was prepared by column chromatography. For separation of torulene isomers, a YMC C_30_ column (5 µm, 250 × 4.6 mm) was used, together with a binary gradient mobile phase consisting of methanol–methyl *tert*-butyl ether-water, (50:47.5:2.5, v/v/v) (A) and methanol–methyl *tert*-butyl ether-water, (8:90:2, v/v/v) (B). In these conditions, eight isomers of torulene, with a molar mass of 535 g/mol, were isolated: di-Z-Torulene (isomers 1 and 2), mono-Z-Torulene (isomers 3–7) and All-*E*-Torulene. It is probable that, due to the presence of 13 double bonds, more torulene *cis* forms are formed.

**Table 2:** Chromatographic analysis methods used for torulene and torularhodin

Mobile phase composition	Elution type	Analytic column type	Detector	Literature
Acetonitrile:isopropanol:ethyl acetate 4:4:2 (v/v/v)	Isocratic	C18 (Restek Ultra type, Restek)	Evaporative light scattering detector	[94]
Acetonitrile:dichloromethane:methanol 7:2:1 (v/v/v)	Isocratic	Spherisorb ODS2 (Alltech Associates)	Refractometer detector	[95]
Acetonitrile:tetrahydrofuran:water 5:3:1 (v/v/v)	Isocratic	C18 (µ-Bondapak type, Waters)	UV/Vis detector; 501 nm	[91]
Methanol:acetonitrile 9:1 (v/v/v)	Isocratic	C18 (Novapak C type, Waters)	UV/Vis detector; 450 nm	[96]
Acetone:water 95:5 (v/v)	Isocratic	RP-18 (LiChrospher 100 type, Merck)	UV/Vis detector; 450 nm	[97]
Acetonitrile:methanol:methylene chloride 71:22:7 (v/v/v)	Isocratic	C18 (Waters type, Milford)	UV/Vis detector; 420–500 nm	[98]
A: acetone with a 0.1% addition of trifluoroacetic acid (TFA) B: water with 0.1% TFA	Gradient	RP-18 (LiChrospher 100 type, Merck)	Diode-array detector	[27]
A: methanol: ethyl acetate 1:1 (v/v) with a 0.05% addition of triethylamine and 0.1% BHT B: acetonitrile with a 0.05% addition of triethylamine and 0.1% BHT	Gradient	C18 (Supelcosil type, Sigma-Aldrich)	UV/Vis detector; 450 nm	[85]
A: acetone B: water	Gradient	C18 (Cosmosil type, Nacalai Tesque)	Diode-array detector	[99]

## Suggested procedure for industrial production of torulene and torularhodin

**Fig. 4:**
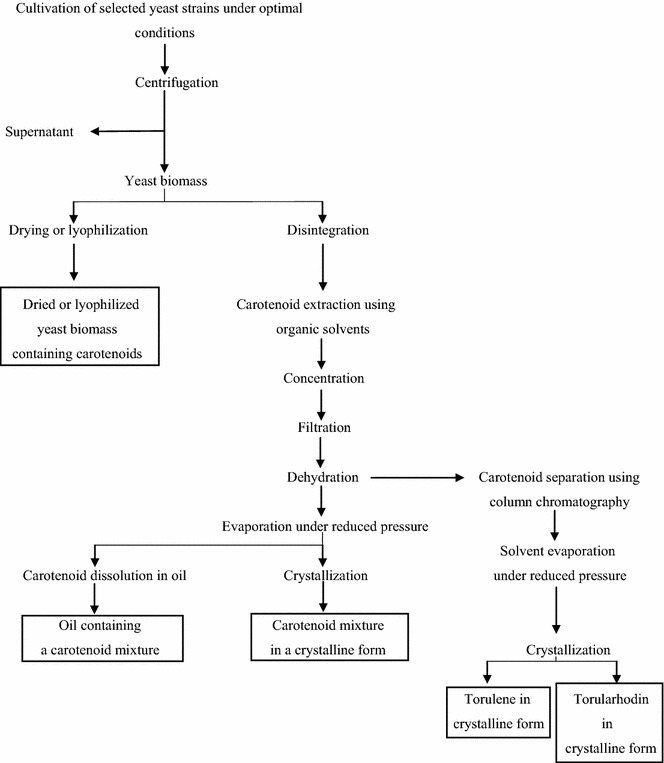
Suggested diagram for the production of torulene and torularhodin using yeasts [prepared on the basis of works of 16, 78, 100–105]

Torulene and torularhodin are not currently produced or used in industry; however, they have many potential applications thanks to their properties. A suggested production diagram for torulene and torularhodin using yeasts is presented in Fig. 4, prepared on the basis of information on carotenoids included in the literature [16, 78, 100–105]. Obtaining satisfactory production yields requires appropriate yeast strains characterized by intensive carotenoid synthesis to be selected, as well as optimal conditions and microbial cultivation parameters to be determined. After cultivation, the first stage of the process includes separation of yeast biomass from the post-culture medium, e.g., using centrifugation [106]. Thus, obtained yeast biomass may be subsequently dried or lyophilized [107], and the obtained product may be used as a valuable additive to feedstock for animals [53]. Separation of torulene and torularhodin from yeast cells requires an appropriate disintegration method, allowing efficient extraction of these dyes [78]. In the next stage, the extracted carotenoids dissolved in organic compounds may be subjected to individual operations such as concentration, filtration, and dehydration [108]. Next, it is necessary to evaporate the residues of organic solvents under reduced pressure, which guarantees stability of the dyes [103]. The evaporation residue may be subsequently dissolved in various edible oils, thus obtaining an oil enriched with a mixture of carotenoids [109] or crystallized in order to separate the mixture of dyes in its crystalline form [100]. In order to obtain the torulene and torularhodin fractions, it is necessary to separate the obtained carotenoid extract using chromatography, and the separated fractions may be subsequently crystallized [104] obtaining preparations of torulene and torularhodin.

## Properties and potential applications of torulene and torularhodin

Torulene and torularhodin are absent in food, thus their influence on the human body has not been studied and described as yet. However, taking into account their chemical structure and properties, these compounds may probably be successfully used as food additives and additives in feedstock for animals, and in cosmetics [16] as well as ingredients in medicines [14]. Research on properties and potential use is presented below.

### Toxicity

Latha and Jeevaratanm [110] studied the toxicity of carotenoids produced by *R. glutinis* DFR-PDY yeasts in rats. This strain synthesizes β-carotene, torulene, and torularhodin, as described by the authors in regard to their previous studies [88]. Over the course of 13 weeks, animals received a lower (100 mg/kg of body mass) and a higher (300 mg/kg body mass) dose of carotenoids. The control group animals received only palm oil. A blood count examination did not indicate significant differences in the count of red blood cells, the hematocrit number, platelet count, mean corpuscular volume (MCV), or mean corpuscular hemoglobin concentration (MCHC), whereas the count of white blood cells was significantly lower only in males that received the higher dose of carotenoids. Urine parameters of experimental animals did not differ significantly in the control group and in the groups consuming the lower and the higher carotenoid doses. Histopathological examinations of lungs, liver, and kidneys did not show pathological changes in the tested rat groups. The authors concluded that carotenoids synthesized by the *R. glutinis* DFR-PDY yeasts may be used as food additives, which infer anti-oxidative properties in addition to their colorant role [110].

### Anti-cancer activity

Another study [111] tested anti-cancer activity of carotenoids synthesized by *R. glutinis* NCIM 3353 yeasts in male Wistar race rats. Powdered biomass of *R. glutinis* yeasts containing torulene, torularhodin, and β-carotene in a 58:33:6 ratio was added to the feedstock. On the basis of the obtained results the authors concluded that carotenoids showed protective properties in preneoplastic changes of liver induced by dimethylnitrosamine. Du et al. [112] tested the anti-cancer activity of carotenoids in mice that orally received 9 or 18 mg/kg/day of torulene or torularhodin, respectively. After 2 weeks of supplementation, the mice were infected with hormone-independent PC-3 prostate cancer cells and the administration of carotenoids was continued. The animals were euthanized after 7 weeks and subjected to histopathological analysis. These examinations showed that both torulene and torularhodin significantly inhibited the development of prostate cancer in the studied mice. Diet supplementation with torulene and torularhodin at 18 mg/kg body mass resulted in a decrease in tumor volume from 248.13 ± 28.74 to 70.34 ± 6.77 and 60.53 ± 6.78 mm^3^, respectively. This effect was related to apoptosis of cancer cells (reduction of Bcl-2 proteins) and amplified expression of the Bax protein and of caspase-3, -8, and -9. The next work [113] confirmed the anti-cancer properties of torulene using prostate cancer cells of the LNCaP line. Wu et al. [114] concluded that torularhodin shows neuroprotective activity against H_2_O_2_—induces oxidative injury, related to its strong anti-oxidative activity.

### Anti-oxidative properties

Both torularhodin and torulene exhibit strong anti-oxidative properties. Dimitrova et al. [29] determined the anti-oxidative capacity (ORAC) of these two compounds using Trolox. Values of this indicator for torulene and torularhodin were 2.77 and 3.9, respectively, and were lower than anti-oxidative capacity determined for β-carotene (3.78). Other studies [18] noted that torularhodin neutralizes free radicals more efficiently than β-carotene. Oxidation reaction of 1,3-diphenylisobenzofuran (DPBF) was used for the purpose, with 3-(1,4-epidioxy-4-methyl-1,4-dihydro-1-naphthyl)propionic acid (EPA) as a source of singlet-state oxygen. Under such conditions, DPBF decomposed at a slower rate (0.002 mM/h) than in a sample using β-carotene (0.013 mM/h), which led to a conclusion that torularhodin neutralizes free radicals more efficiently than β-carotene. Ungureanu and Ferdes [14] determined anti-oxidative activity of methanolic torularhodin extracts using chemiluminescence. The studied extracts showed strong anti-oxidative activity. Torularhodin showed the activity of 96% directly after extraction, and this value decreased by 7 and 17%, respectively, after 7 and 14 days. Anti-oxidative capacity was determined simultaneously using photochemiluminescence, and the value of this index was found to be 255.6 µg/mL of Trolox equivalents.

### Anti-microbial activity

Carotenoids synthesized by yeasts of the *Rhodotorula* and *Sporobolomyces* genera show strong anti-microbial activity [14, 115–117]. Ungureanu and Ferdes [14] determined the anti-microbial activity of methanolic torularhodin extracts. The performed study led to the conclusion that torularhodin showed anti-bacterial and anti-fungal properties toward all tested strains. The growths of *Fusarium oxysporum* MUCL 791 and *Aspergillus ochraceus* molds and *Candida utilis* yeasts and *Enterococcus faecalis* ATCC 29212 bacteria was inhibited at a torularhodin concentration of 44.375 µg/L. *Escherichia coli* K 12-MG1655 and *Staphylococcus aureus* ATCC 25923 bacteria were more susceptible to the activity of this compound, and the MIC value was 22.18 µg/L. The strong anti-microbial properties of torularhodin may also be used in the production of implanted medical products. Although stringent sterile procedures are observed, peri-implantation infections interrupting the process of implant connection to the living tissue are still being diagnosed. Microorganisms may colonize surfaces of materials, devices, and medical implants, forming biofilms [118]. Ungureanu et al. [119] determined the properties of titanium implants coated with a torularhodin solution, with an additional layer of titanium dioxide used. Titanium implants were dipped in a torularhodin solution (5 mg/L) or in a 1:1 mixture of dopamine (5 × 10^−3^ M) and torularhodin. Anti-microbial activity was tested on both Gram-negative (*E. coli* ATCC 8738, *Pseudomonas aeruginosa* ATCC 9027) and Gram-positive bacteria (*S. aureus* ATTC 25923, *E. faecalis* ATCC 29212, *Bacillus subtilis* ATCC 6633). Torularhodin effectively inhibited proliferation and adhesion of the studied bacterial strains, and the anti-microbial activity of films containing torularhodin and torularhodin with dopamine was higher than that of cefotaxime (III generation antibiotic) toward *E. coli*, *S. aureus* and *B. subtilis* bacteria. Moreover, titanium implants with an additional coating were characterized by good hemocompatibility, allowing them to be potentially used in the production of medical products.

## Conclusions and future prospects

Torulene and torularhodin belong to a group of carotenoids synthesized only by yeast and fungi. These compounds are not currently produced on an industrial scale, however, they may be potentially used in many sectors of industry through to their valuable properties. First, these compounds can be used in the food industry and animal feed. Torulene and torularhodin could be used as antioxidants, as well as dyes, due to their rosy-red color. So far, the properties of these compounds have been only tested in rats and mice, so further research should required to determine their influence on the human body. It is also necessary to extend the toxicity studies and to determine the dose of ADI. The second direction concerns the use of torulene and torularhodin in the production of implanted medical products. Studies have shown that these compounds have strong anti-microbial properties, particularly against *E. coli* and *S. aureus*. Due to these properties, films containing these compounds can effectively protect against peri-implantation infections. The third direction concerns the use of torulene and torularhodin in the prevention of tumors, especially prostate cancer. The results are promising, but the scope of the research should be extended. In addition, another direction of studies should be aimed at the use of the waste microorganisms biomass, which may be used as feedstock additive for animals after extraction and removal of solvents. The use of waste biomass, rich in proteins and polysaccharides, would additionally improve the profitability of microbial carotenoid production.

Serious obstacle to the commercialization of production torulene and torularhodin is the low efficiency of biosynthesis. At present, profitable method of production has not been developed and high-yield producers of these compounds have not been obtained. The problem is also that the main producers of these compounds are yeasts that do not have GRAS status. Solutions to this problem can be found in modern genetic engineering techniques. If high-yield safe yeast mutants were to be obtained and the costs of producing torulene and torularhodin were low, these compounds would probably have been used in various industries.
